# A two-tiered curriculum to improve data management practices for researchers

**DOI:** 10.1371/journal.pone.0215509

**Published:** 2019-05-01

**Authors:** Kevin B. Read, Catherine Larson, Colleen Gillespie, So Young Oh, Alisa Surkis

**Affiliations:** 1 NYU Health Sciences Library, NYU Langone Health, New York, New York, United States of America; 2 Institute for Innovations in Medical Education, NYU Langone Health, New York, New York, United States of America; Columbia University, UNITED STATES

## Abstract

**Background:**

Better research data management (RDM) provides the means to analyze data in new ways, effectively build on another researcher’s results, and reproduce the results of an experiment. Librarians are recognized by many as a potential resource for assisting researchers in this area, however this potential has not been fully realized in the biomedical research community. While librarians possess the broad skill set needed to support RDM, they often lack specific knowledge and time to develop an appropriate curriculum for their research community. The goal of this project was to develop and pilot educational modules for librarians to learn RDM and a curriculum for them to subsequently use to train their own research communities.

**Materials and methods:**

We created online modules for librarians that address RDM best practices, resources and regulations, as well as the culture and practice of biomedical research. Data was collected from librarians through questions embedded in the online modules on their self-reported changes in understanding of and comfort level with RDM using a retrospective pre-post design. We also developed a Teaching Toolkit which consists of slides, a script, and an evaluation form for librarians to use to teach an introductory RDM class to researchers at their own institutions. Researchers’ satisfaction with the class and intent to use the material they had learned was collected. Actual changes in RDM practices by researchers who attended was assessed with a follow-up survey administered seven months after the class.

**Results and discussion:**

The online curriculum increased librarians’ self-reported understanding of and comfort level with RDM. The Teaching Toolkit, when employed by librarians to teach researchers in person, resulted in improved RDM practices. This two-tiered curriculum provides concise training and a ready-made curriculum that allows working librarians to quickly gain an understanding of RDM, and translate this knowledge to researchers through training at their own institutions.

## Introduction

Better data management on the part of researchers is recognized as a critical need by researchers, funders, and publishers [1–3]. Good research data management (RDM) practices provide the means to analyze data in new ways, more effectively build on another researcher’s results, reproduce the results of an experiment, and aggregate like datasets for analysis [4–6]. While the benefits of RDM are clear, researchers often overlook the importance of RDM throughout the research process. The reasons for this are well-documented [7–10]: researchers see no benefit to themselves in exercising good RDM practices, they do not believe anyone would want or be able to understand their data, grant and publication pressures leave them no time, and there is no money to support RDM. The goal of this project was to facilitate better RDM on the part of researchers through the development of concise online modules to provide librarians with the knowledge and comfort level to teach RDM, and a ready-made, flexible curriculum for librarians to use for training researchers at their own institutions.

Librarians, with their knowledge of metadata, preservation, and discovery, are recognized by many as a potential resource for assisting researchers with RDM [11–18]. However, this potential has not been fully exploited, particularly in the biomedical research community. While this is in part due to institutional barriers and the failure of researchers to recognize librarians’ expertise in this area, a major barrier to fulfilling this potential lies with the shortcomings of resources available to librarians. Before embarking on this project, the authors disseminated a survey to health sciences librarians through professional listservs asking respondents if they saw a role for their library in teaching RDM, if they currently taught RDM and, if not, what they saw as barriers to doing so [19]. There were 118 survey responses, with 84% of respondents indicating they saw a role in teaching RDM at their institutions, but 75% indicating that they did not currently do so. Barriers identified included a lack of knowledge about RDM (60%), lack of comfort engaging with researchers around the topic (48%), and lack of satisfactory curricula to train researchers (44%) were barriers to supporting their own research communities.

While web-based RDM educational modules for librarians already exist [20–22], until recently [23] none have had a biomedical focus or addressed librarians’ familiarity with the research process. Many of these online educational offerings require a more substantial time commitment [22, 23], which can be a significant barrier for working librarians. Another gap has been the lack of a ready-made, biomedically-focused RDM curriculum for use by librarians in training researchers. To fill the gaps that exist in available RDM training, we developed and piloted two curricula: 1) a web-based curriculum that teaches health sciences librarians about RDM, data, researchers and the biomedical research process [24] and 2) a toolkit consisting of slides, script, instructions, and an evaluation form for an introductory RDM class [25].

## Materials and methods

### Recruitment

The intent of the pilot project was to train health sciences librarians and provide them with the tools to teach RDM to researchers, specifically within the context of the research landscape in the United States (US). The criteria for pilot participation was therefore that learners be a) health sciences librarians, and b) working at an institution in the US. Recruitment of pilot participants was completed through emails to health sciences librarian listservs and newsletters disseminated through the National Network of Libraries of Medicine (NNLM). The platform that hosted the online modules was freely accessible, therefore the modules could be taken by anyone. As a result, learners who had not been directly targeted as part of the pilot recruitment process and who did not fit the pilot criteria found and took the modules. Because we had not anticipated this, none of the assessment questions embedded in the online modules directly assessed learners’ suitability for the study. We therefore filtered out the following from our initial sample (n = 89): 1) non-librarian users at any NYU domain (n = 16), 2) users at any non-US domain (n = 4), and 3) users whose free-text comments indicated they were not US-based health sciences librarians (n = 4). We also removed duplicate users (n = 2), keeping only their first response. The number of librarians completing each module and the number of institutions represented by those librarians is listed in Table 1.

**Table 1:** Number of module completions by librarian and by institution.

Module	Number of librarians completing module	Number of institutions completing module
The Story of Data	63	46
The Data Lifecycle	55	42
Understanding Researchers	42	31
Research Data Management Climate	33	25
Data Documentation Best Practices	30	23
Data Standards	29	22
Storage, Preservation, and Sharing	28	21
Assessment at completion of all modules	27	20

The piloting of the Teaching Toolkit consisted of the project Principal Investigators (PIs) completing site visits to observe librarians using the Teaching Toolkit to teach an in-person class for researchers at their institution. Eligible librarians were those who had completed all seven modules and whose responses in the completion survey indicated that there was a possibility of them teaching within the timeframe of the grant. There were 18 librarians from 15 institutions who met this criteria. Three of these institutions were able schedule an RDM course within the timeframe of the grant, and therefore all three were selected for piloting.

### Online modules

We created seven web-based modules for librarians, utilizing content based on our experience teaching RDM to librarians and researchers and designing the modules based on the cognitive science of learning theories to enhance educational effectiveness [26–30]. The modules were published online using a platform developed at the NYU School of Medicine that allows for authoring, dissemination, and data collection of web-based learning modules [24]. Initially, we created seven modules, with the order, content, and objectives of those modules mirroring existing classes the project PIs taught to researchers starting in 2012 and health sciences librarians starting in 2014 as seen in Table 2.

**Table 2:** Online research data management education modules for librarians.

Module Title	Module Description	Module Learning Objectives
The Story of Data	Background information to provide a concrete understanding of the different forms that research data can take and data pathways from conceptualization to collection to processing to analysis.	• Distinguish between research data management needs of different categories of data • Identify the full range of data products that should be recorded for a study • Distinguish between raw, processed, and analyzed data
The Data Lifecycle	Introduction to the research data lifecycle as a structure for mapping out the full range of data management activities, and how they align with the research process.	• Pinpoint the data management needs at each stage of the data lifecycle • Distinguish between data management needs for reproducibility purposes versus reuse • Identify different options for researchers to disseminate their data
Understanding Researchers	Description of the differences between bench and clinical research processes, environment, and data management needs and issues.	• Identify differences between research practices of bench science and clinical research • Identify data management issues in bench science and clinical research
Research Data Management Climate	Incentives, requirements, and associated expectations that illustrate RDM’s importance within biomedical research.	• Recognize requirements that enforce the managing and sharing of research data • Identify incentives that will encourage researchers to manage and share their data • Use resources that will facilitate researchers managing and sharing their data
Data Documentation Best Practices	Introduction to basic concepts of effective data management through discussion of workflow, file naming conventions, and best practices in variable names.	• Outline all the components of a research workflow that should be documented • Apply best practices for file naming • Document variable names in a data dictionary • Apply best practices to variable selection and naming
Data Standards	Introduction to discipline-specific data standards, and explanation of their importance in collecting data and providing metadata for research data	• Recognize the value of using standards for research • Locate standards for various biomedical disciplines • Distinguish between terminologies, reporting guidelines, and data models as standard types
Storage, Preservation, and Sharing	Methods for researchers to effectively store, archive and preserve their data.	• Select the appropriate storage solution(s) for datasets • Communicate the difference between storage and preservation • Evaluate repositories and assess the pros and cons for sharing different types of data

The modules included videos, text, and embedded questions to assess the following: 1) users’ experience of the modules, 2) changes in self-reported understanding and comfort level with the material, and 3) intent to use the knowledge gained. The questions were not drawn from a validated instrument as none exist designed for our purposes. However, the questions were developed in consultation with a collaborator (CCG) with evaluation expertise, adapted from instruments used within our medical school curriculum that have evidence of their reliability (internal consistency) and validity (expected associations among and across evaluation domains) across their use in different contexts (e.g., obtaining feedback on and evaluation of courses, clinical experiences, and online modules), and were pilot-tested and refined with experts and then librarians through several iterative cycles.

The modules could only be accessed in the order listed in Table 2 for the purposes of the pilot. Questions embedded before the first module assessed each librarian’s overall background and interest level in RDM. Questions embedded at the conclusion of each module assessed librarian satisfaction with the module and their self-reported change in understanding. Questions embedded after completion of all modules assessed librarians’ self-reported change in comfort level with the material, plans to teach RDM, and intent to use the material learned in other aspects of their work. The self-reported changes in understanding and comfort level were assessed using a retrospective pre-post design to correct for participants’ tendency to overestimate understanding and confidence at baseline and then re-calibrate more accurately after training [31–34]. A four point scale was used to assess both self-reported understanding (no, minimal, moderate, strong) and self-reported comfort level (not, somewhat, mostly, very). Embedded questions and responses are included in S1 Data and S2 Data.

It was decided that, while not part of the original protocol, a more complete assessment of the strengths and weaknesses of the online modules could be gained through semi-structured telephone interviews with participants who had completed all seven modules. Because of the time required by this additional phase, we selected 14 interviewees, 2 based on their RDM expertise and 12 based on their suitability to be pilot participants for using the Teaching Toolkit at their own institution. Suitability criteria was based on librarians’ self-reported interest in, and intent to use the material in the online modules.

### Teaching Toolkit

We developed a Teaching Toolkit which consisted of slides, a script, and an evaluation form for class attendees [25]. The material was designed to be used to teach a 60 to 90 minute introductory RDM class with the content drawn from the curriculum of the first seven online modules (Table 2). We piloted the Teaching Toolkit with librarians from three institutions. The two project PIs traveled to the institutions to observe the classes being taught and conduct semi-structured interviews with the librarian(s) who had taught the class.

The semi-structured interview (see S1 File) asked the librarians to reflect on the use of the Teaching Toolkit, describe further plans for use of the Teaching Toolkit, describe their professional background, and reflect on the suitability of the Teaching Toolkit for their particular audience. Interviews were designed to elicit feedback from the librarians that would elucidate the strengths and limitations of the Teaching Toolkit’s content, approach, and suitability for a range of instructors and audiences. The interviews were transcribed and a simple content analysis was used to code the major themes that emerged from the interviews regarding the strengths and limitations. Co-authors (KR and AS) discussed codes to ensure agreement and quotes representing identified strength and limitation codes are reported.

Class attendees at the three institutions were asked to evaluate the class (see S2 File), answering questions about their satisfaction with the material and their intent to use what they had learned. Seven months after the class, a follow-up up survey (see S3 File) was sent to researchers to assess whether they had actually used what they learned. Researcher data is included in S3 Data.

The collection of evaluation and interview data was approved by the NYU Langone Health Institutional Review Board.

### Data analysis

We used a Wilcoxon signed-rank test to test the difference between self-reported understanding before and after each module, and between self-reported comfort level before and after the series of modules. In both cases, we used the one-sided test because, having done retrospective pre-post assessments, we did not expect decreases in self-reported understanding or comfort level. We used a normal approximation because of ties in the data. We used a chi-squared test of the effective ratings across the modules to determine if there was any significant differences in the learners’ perceptions of the effectiveness of the different modules. We otherwise used descriptive statistics to characterize responses.

## Results

### Online modules

#### Assessment

**Fig 1:**
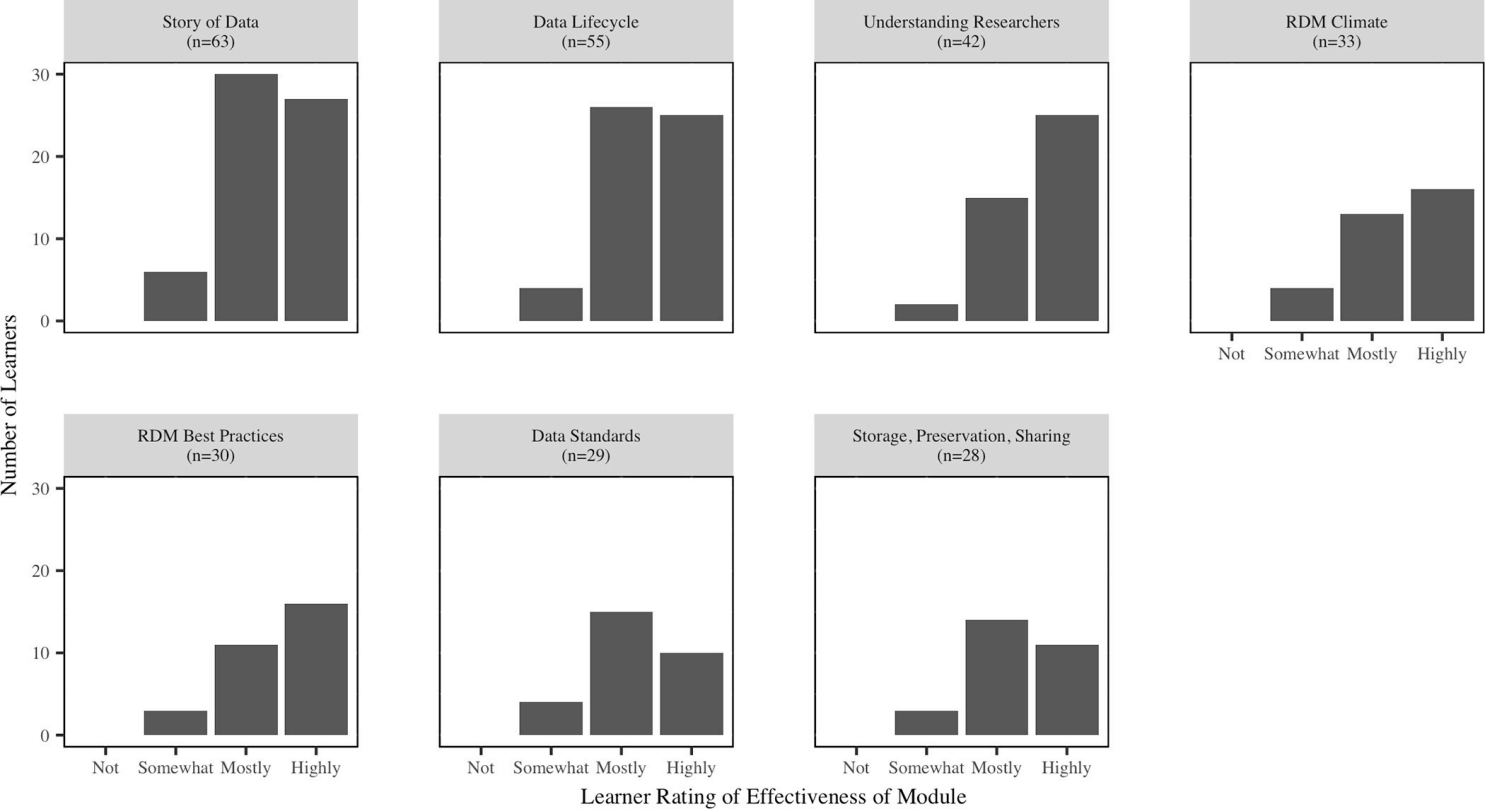
Librarian rating of effectiveness of each of the seven online modules.

Responses to the embedded questions at the completion of each module, indicated that the majority of librarians found the modules to be mostly or highly effective (Fig 1). A chi-squared test of the ratings across the modules indicates that there is no significant difference in effectiveness across the modules (p = 0.18). Across all modules, 91% indicated that they found the level of the material to be “just right” and 91% indicated that the length of the module was “just right”.

**Fig 2:**
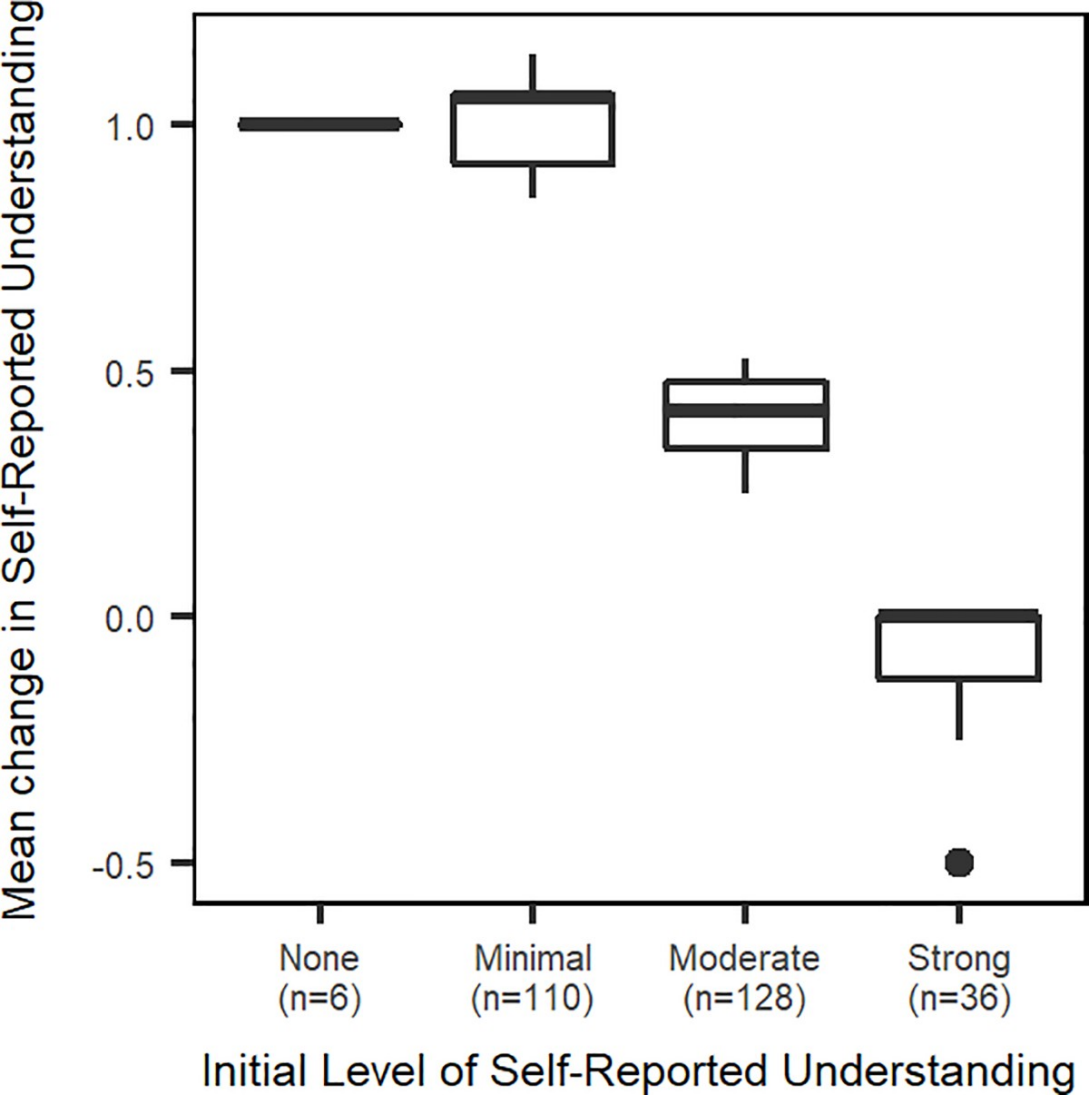
Change in self-reported understanding, categorized by initial level of self-reported understanding, aggregated across modules.

Using the Wilcoxon signed-rank test, we found the differences between self-reported understanding from pre- to post-module to be significant, with p < 0.0005 for each module. The mean difference in level of self-reported understanding varied by level of self-reported pre-understanding, with larger increases in self-reported understanding seen for those who reported having had no or minimal understanding of the content before the module (Fig 2).

**Fig 3:**
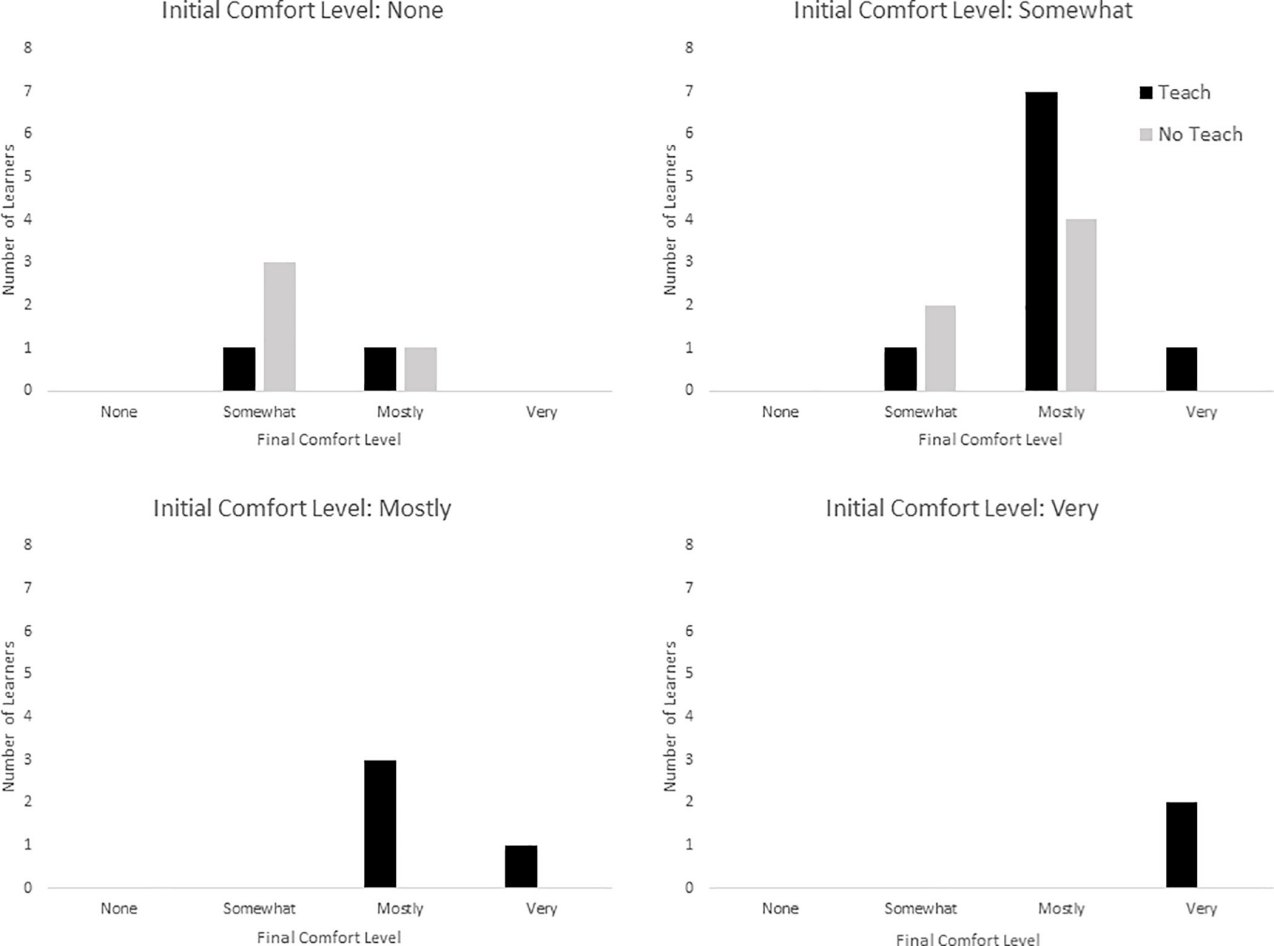
Learner counts of final comfort level with RDM grouped by initial comfort level. For each initial comfort level, final comfort levels are shown, and are grouped by whether or not the learner felt they had sufficient knowledge to teach RDM.

The self-reported comfort level of learners (n = 27) increased from a median of 2 scale points (somewhat comfortable) to a median of 3 scale points (mostly comfortable), and the change in comfort level was seen to be significant using the Wilcoxon signed-rank test (p = 0.0002). Librarians change in self-reported comfort differed by initial comfort level: not comfortable (n = 6) mean increase of 1.33 scale points, somewhat comfortable (n = 15) mean increase of 0.87 scale points, mostly comfortable (n = 4) mean increase of 0.25 scale points, very comfortable (n = 2) mean increase of 0. Fig 3 shows the final self-reported comfort level for each librarian grouped by their initial comfort levels, and indicates which of the librarians felt they had sufficient knowledge to teach at the conclusion of the modules. Finally, 82% of librarians indicated that they would otherwise use what they had learned in their work.

#### Post-module telephone interviews

Telephone interview responses indicated that a common barrier to teaching RDM was a lack of knowledge about strategies for finding institutional avenues for teaching a class. We therefore created an eighth module to provide strategies for libraries to initiate RDM services locally. We discussed likely partners and strategies for implementation and included both those that had been successful in our own institution [10, 18] as well as other institutions [35]. Of those librarians interviewed, three were able to teach within the timeframe of the pilot, and so formed the pilot cohort for the Teaching Toolkit.

### Teaching Toolkit

The Teaching Toolkit was piloted at three health sciences libraries. There were 16 total attendees across the three classes, with 10 of those consenting to the use of their evaluation data. Attendees self-identified as having the following roles: staff (4), faculty (3), postdoc (1), fellow (1), and student (1).

**Fig 4:**
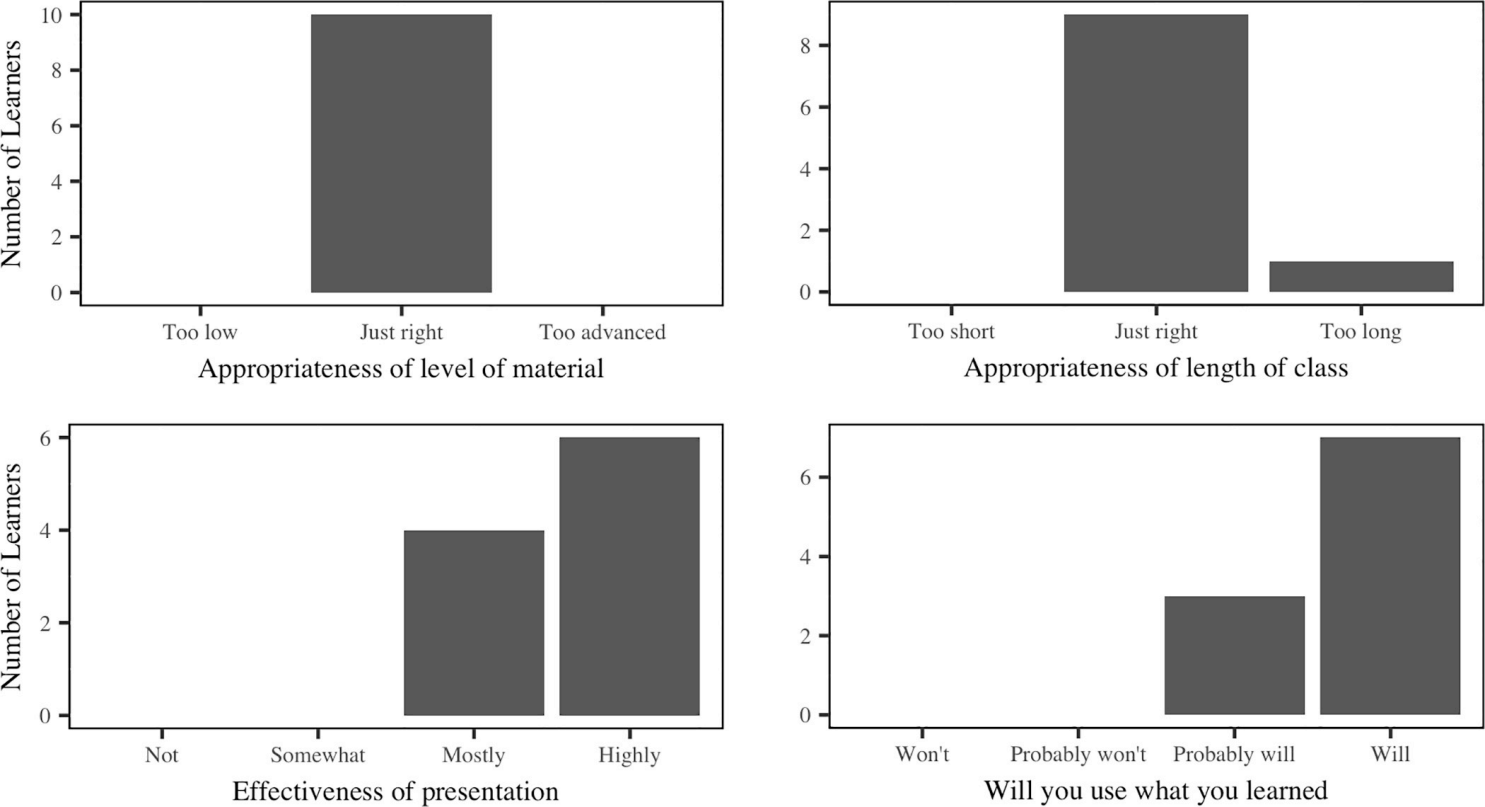
Learner satisfaction reports. Self-reporting of satisfaction with in-person RDM class for the following: level of class, length of class, effectiveness of presentation, whether the learner will use what they learned.

The attendees’ ratings of the level, length, and effectiveness of the class (Fig 4) indicated a high degree of satisfaction with the class. All ten attendees indicated that they had had no previous exposure to RDM educational materials, and all ten indicated they would either definitely or probably use what they had learned (Fig 4). The follow-up survey (see S3 File) administered to the attendees seven months after the class received five responses, and in all cases attendees reported that they used what they had learned. Four of the five responded to a free text question asking for a description of how they had used what they learned in the course, and all four stated that the course had helped them with the organization of their data and three specifically pointed to using best practices for improving file naming conventions.

Our observations of the classes at each institution documented a range of presentation styles, with customizations made to the script and the slides. One instructor added more interactive elements to the class to encourage participation. Each pilot institution made visual modifications to the slides provided, added institutional resources for data storage and sharing, promoted library services around data management, and added examples of data management best practices to make the slides more engaging and better suited to their personal style.

Observing the in-person classes provided an opportunity to identify topics of particular interest to the audience, as well as elements of the presentation that could be improved upon. For example, all of the instructors had some level of discomfort with the material related to teaching the NIH rigor and reproducibility requirements. Based on this observation, we modified the content of the slides and script to streamline and clarify this component of the class. In addition, we made a number of other minor modifications to improve the clarity and flow of the class. We observed that the class attendees were the most engaged during the sections of the curriculum that discussed data organization, preservation, and standards.

Semi-structured interviews (see S1 File) conducted with the instructors (n = 4) from each pilot institution yielded a number of common themes. All instructors discussed the challenge of using material that they had not created. Instructors mentioned difficulties connecting with the material, specifically citing discomfort in the use of a script they had not written themselves. Despite the lack of comfort with the script, instructors felt strongly that the classes were successful, which was confirmed by the evaluation data (Fig 4). Pilot instructors all mentioned a desire to add interactive elements to the class. Instructors also stated that they saw value in all of the material included in the Teaching Toolkit, and did not plan to remove any of the topics discussed.

## Discussion

The curricula we developed provided significant innovation over existing data management education modules [20, 21, 36] in several areas. Our online modules were aimed at librarians and focused on biomedical research. These online modules included training on the processes, data, culture, and language of biomedical research to provide critical context that would allow librarians to overcome the barriers between librarians and researchers. The online modules were concise and directly tied to the Teaching Toolkit, a curriculum specifically created for use by the librarians to teach RDM locally, thus addressing the time constraints of working professionals seeking to enter this area.

One limitation of this study was the sample size, both for librarians taking the online modules and for researchers attending the Teaching Toolkit class. While the sample size of librarians would be low for online educational modules aimed at the general population, our modules were intended for the very specific population of U.S. based health sciences librarians. A rough estimate of the number of potential participants in this pilot is 300 (157 academic health sciences libraries, typically with no more than two librarians engaged in research data management services). Given that, our sample size of 63 librarians for the initial module and 27 librarians completing all modules constitutes approximately 20% and 10% respectively of the total population on which we were drawing. The small sample size of researchers attending the Teaching Toolkit classes is more problematic, and limits any generalizations that can be made based on that data. However, the unanimity of responses is encouraging, and results from a second pilot project by the authors, discussed later, support the conclusion that the Teaching Toolkit is effective and generalizable.

Online modules have been seen to have a high dropout rate; reported in multiple studies to be 90% or higher [37–39]. We saw a 57% dropout rate between online modules 1 and 7. This dropout rate is low relative to what has been reported in the literature, but we acknowledge that it may have introduced bias into our results. We have some evidence that argues against this; during the pilot we conducted a survey of librarians who had expressed a strong interest in RDM but dropped out of the online modules. The two question survey (see S4 File) asked respondents to report the reason(s) for their non-completion, and elaborate on those reasons. The survey was distributed to 25 librarians and received 13 responses, 92% of whom indicated lack of time as one reason for non-completion, and for 62% of respondents, lack of time was the only reason indicated. The only other reason provided by more than one respondent was that it was not applicable to the respondents’ work (23%). These results were not unexpected since our target population was working librarians, who often must fit in professional development on their own time.

Another limitation is that we have no direct measurement of knowledge gain, since during the piloting process, a number of issues were uncovered in the modules’ knowledge gain questions, rendering that data unusable. We are therefore forced to rely on self-report of change in understanding of subject matter, however, our primary outcome was the librarians’ ability to teach an RDM class, not their overall knowledge of RDM.

A strength of this study is that the online modules proved to be effective in increasing librarians’ self-reported understanding of and comfort level with RDM. The increase in self-reported understanding for those with little or no understanding of the topic was greater than for those with previous experience. This outcome is expected as the modules were created to provide a concise introduction to RDM, rather than a deep dive. Future RDM education for librarians could modularize topics based on discipline, to provide training on targeted issues faced by specific research communities. The expressed need from the 14 phone interviews for additional guidance on initiating RDM services indicated that providing the knowledge and tools needed to teach a class only partially addresses the needs of librarians looking to provide services in this area.

Another strength of the study was that the Teaching Toolkit showed promise as an effective educational intervention; all respondents from the in-person class evaluation indicated that they would probably or definitely use what they had learned in their work, though the small sample size (n = 10) limits the conclusions we can draw. The positive reviews of the in-person class are particularly striking in light of the discomfort expressed by the instructors at teaching material that was not prepared in their own voice. Despite the awkwardness reported with use of the script, all instructors indicated that it was useful to have as a starting point. It is likely that the effectiveness will increase as librarians continue to customize the material to reflect their own teaching styles.

While the low attendance was a limitation, we do not believe this reflects an issue with the curriculum. In our own institution we have seen attendance at RDM classes grow from a low of three attendees in 2014 to over 100 signups for an RDM class in 2018. As guidelines and mandates from publishers and funders further increase the pressure on researchers to employ good RDM practices, and as the role of the library in supporting RDM is increasingly recognized within institutions, we would expect that class attendance would increase at other institutions as it has at ours. To this point, our pilot institutions have since reported additional trainings with much higher attendance.

Further validation of the value of our materials to the library community has come through two avenues. First, six of the eight online modules were used as a core component of a training program developed by the National Network of Libraries of Medicine (NNLM) titled *Biomedical and Health Research Data Management for Librarians*, which recruited 40 librarians from across the U.S. to take a comprehensive data management training over an eight-week period [40]. Second, both the online modules and the Teaching Toolkit formed the core of a subsequent NNLM-funded pilot project led by the PIs to facilitate the development of RDM services in libraries.

The NNLM-funded pilot project enrolled 26 librarians from six libraries [41]. This pilot resulted in every institution using the Teaching Toolkit to teach between one and five classes with a total of 294 attendees (average attendance = 21), where 99% of those who submitted an evaluation (n = 111) indicated that they would use what they had learned, and 95% (n = 112) indicated that they would recommend the class. Between the NNLM-funded pilot project and the project described in this manuscript, seventeen classes were taught across eight institutions (five health sciences libraries, three university libraries), to over 300 attendees from a wide range of backgrounds (e.g., health sciences, engineering, social sciences). These results indicate that the curriculum is broadly generalizable across a range of academic contexts and audiences.

The online modules and Teaching Toolkit provide effective, approachable RDM training and a ready-made, flexible, proven curriculum. The goal of our minimalist approach is to provide just enough background knowledge to equip librarians to provide effective trainings for researchers in the essentials of RDM, as well as sufficient context about researchers and the research process to increase the librarians’ comfort in engaging with researchers. This approach meets a critical need for practicing librarians with limited time who are seeking to initiate RDM services. While this does not provide the depth of RDM knowledge of more extensive curricula, we believe that researcher engagement resulting from teaching RDM classes is the most effective driver for working librarians to develop deeper RDM expertise. With the help of our curricula, institutions in search of a resource for supporting data management in their research community can look to their library for effective support.

## Supporting information

S1 Data Librarian self-reported satisfaction with and knowledge gained from each of the seven online modules. (CSV)

S2 Data Librarian self-reported satisfaction, change in comfort level and intent to use at the conclusion of all online modules. (CSV)

S3 Data Researcher self-reported satisfaction and intent to use material at the conclusion of the class taught by librarians using the Teaching Toolkit, and researcher report of actual use of material seven months post class. (CSV)

S1 File Semi-structured in-person interview form conducted after the completion of the in-person RDM class taught by pilot participants. (PDF)

S2 File Evaluation form administered to attendees/researchers at the completion of the in-person RDM class to gauge satisfaction with the material and their intent to use what they had learned. (PDF)

S3 File Follow up survey administered to attendees/researchers seven months after the in-person RDM class to ask how they used what they learned in their work. (PDF)

S4 File Survey administered to librarians who did not complete all seven online modules to ask reasons why they were not able to complete the entire curriculum. (PDF)
