# Supplementary material for: A two-tiered curriculum to improve data management practices for researchers
Source: PLoS One. 2019 May 1;14(5):e0215509. doi: 10.1371/journal.pone.0215509 (PMC6493725; doi:10.1371/journal.pone.0215509)
Supplement: S1 File — (PDF) [file pone.0215509.s004.pdf]

Semi-structured interview

1. Teacher's overall perceptions of the class
    a. How effective did you feel the teaching materials were?
    b. Do you feel this was the right amount of time to teach this material effectively?
    c. Did you feel that the material was at the right level for this audience?
2. Teacher's perceived comfort level (ease of use) with the material presented
3. What were your concerns with the material before you taught the session?
4. Now that the session is over, can you reflect on those concerns or have other concerns arisen?
5. Did you feel that this was the right audience for the teaching material?
    a. If not, who would be a more appropriate audience?
6. If teaching again, would they present the material in the same way?
    a. If not, how would they change it?
    b. Would you add or adapt any material to be better suited to your local environment?
7. Background of teachers -- experience with audience and teaching
8. Questions that arose in class that require follow up with us?
    a. Questions that arose from students that they want further clarification on?
    b. Questions that occurred to them while they were teaching?
9. What is next for data services in general for you, including further classes?
