# Supplementary material for: A two-tiered curriculum to improve data management practices for researchers
Source: PLoS One. 2019 May 1;14(5):e0215509. doi: 10.1371/journal.pone.0215509 (PMC6493725; doi:10.1371/journal.pone.0215509)
Supplement: S2 File — (PDF) [file pone.0215509.s005.pdf]

Research Data Management Initial Survey

Research Data Management Consent Form
You are invited to take part in an evaluation of this course as part of an NIH-funded research study to assess material for teaching research data management (RDM) to researchers. Your participation will consist of two surveys, each of which will take less than 5 minutes and completed online at your computer or mobile device. The two surveys will be delivered at the end of the course, and 6-months after the completion of the course. There are no known risks or discomforts associated with this evaluation. Your participation will improve educational materials in RDM for researchers. Taking part in this evaluation is completely voluntary. If you choose to begin this evaluation form you can withdraw at any time without repercussion. If you do not wish to participate in the research study, you are still welcome to participate.

Your responses will be kept strictly confidential, and digital data will be stored in secure computer files at the NYU Langone Medical Center. Any report of this research that is made available to the public will not include your name or any other individual information by which you could be identified. If you have questions or want a copy or summary of the study results, you can contact the researcher at the email address above. If you have any questions about whether you have been treated in an illegal or unethical way, contact the NYU Langone Medical Center Institutional Research Board by phone at 212-263-4110 or by email at irb.office@nyumc.org. Please feel free to print a copy of this consent page to keep for your records.

Checking the YES button below indicates that you are 18 years of age or older, and indicates your consent to participate in this survey.

Do you consent to participate in this survey?

- ❏ Yes
- ❏ No

Course Evaluation

Please provide your name: _______________________________

Please provide your email address: _____________________________

Please indicate your school/department/division: ________________________

What is your role at the institution:

- ❏ Staff
- ❏ Student
- ❏ Lab manager
- ❏ Resident
- ❏ Postdoc
- ❏ Fellow
- ❏ Faculty
- ❏ Other: ____________________________

What are the three main points that you can take away from this class?

Will you use what you learned in this class for your work?

- ❏ Definitely will
- ❏ Probably will
- ❏ Probably won’t
- ❏ Definitely won’t

How will you use this information for your work?

Have you participated in similar training/lectures/sessions on data management topics?

- ❏ Yes
- ❏ No

Would you recommend this class to others?

- ❏ Highly recommend
- ❏ Recommend
- ❏ Recommend with reservations
- ❏ Not recommend

Was the level of material presented:

- ❏ Too low
- ❏ Just right
- ❏ Too advanced

Was the material effectively presented?

- ❏ Very effectively presented
- ❏ Mostly effectively presented
- ❏ Somewhat effectively presented
- ❏ Not effectively presented

Was the length of time allotted for this topic:

- ❏ Too short
- ❏ Just right
- ❏ Too long

What was NOT covered today that you would like to see covered in the future?

Please share any additional comments about the class:

