# Supplementary material for: A two-tiered curriculum to improve data management practices for researchers
Source: PLoS One. 2019 May 1;14(5):e0215509. doi: 10.1371/journal.pone.0215509 (PMC6493725; doi:10.1371/journal.pone.0215509)
Supplement: S3 File — (PDF) [file pone.0215509.s006.pdf]

Research Data Management Class Follow-up Survey

Please complete the survey below.

Have you used what you learned in the Perspectives in Research Data Management class in your work to improve your data management practices?

- ❏ Yes
- ❏ No

(if yes) Briefly describe:

(if no) Why not?

