# Supplementary material for: A two-tiered curriculum to improve data management practices for researchers
Source: PLoS One. 2019 May 1;14(5):e0215509. doi: 10.1371/journal.pone.0215509 (PMC6493725; doi:10.1371/journal.pone.0215509)
Supplement: S4 File — (PDF) [file pone.0215509.s007.pdf]

R25 Research Data Management Follow Up

Start of Block: Default Question Block

Q1 What are the **primary** reasons why you did not complete the full 7 research data management modules? Select all that apply:

- ▢ Did not find the material informative (1)
- ▢ Did not like the way the material was presented (2)
- ▢ I was not able to find the time to complete them (3)
- ▢ Material was not applicable to my work (4)
- ▢ Had issues with the online education platform (5)
- ▢ Other, please specify (6) ____

Q2 Please elaborate on why you did not complete the research data management modules:

____

____

____

____

____

End of Block: Default Question Block
